# Social Vulnerability Index and All-Cause Mortality After Acute Ischemic Stroke, Medicare Cohort 2020-2023

**DOI:** 10.1016/j.jacadv.2024.101258

**Published:** 2024-09-06

**Authors:** Xin Tong, Susan A. Carlson, Elena V. Kuklina, Fátima Coronado, Quanhe Yang, Robert K. Merritt

**Affiliations:** Division for Heart Disease and Stroke Prevention, National Center for Chronic Disease Prevention and Health Promotion, Centers for Disease Control and Prevention, Atlanta, Georgia, USA

**Keywords:** acute ischemic stroke, all-cause mortality, COVID-19, hospitalizations, Social Vulnerability Index

## Abstract

**Background:**

Inequities in stroke outcomes have existed for decades, and the COVID-19 pandemic amplified these inequities.

**Objectives:**

This study examined the association between social vulnerability and all-cause mortality among Medicare beneficiaries hospitalized with acute ischemic stroke (AIS) during COVID-19 pandemic periods.

**Methods:**

We analyzed data on Medicare fee-for-service beneficiaries aged ≥65 years hospitalized with AIS between April 1, 2020, and December 31, 2021 (followed until December 31, 2023) merged with county-level data from the 2020 Centers for Disease Control and Prevention/Agency for Toxic Substances and Disease Registry’s Social Vulnerability Index (SVI). We used a Cox proportional hazard model to examine the association between SVI quartile and all-cause mortality.

**Results:**

Among 176,123 Medicare fee-for-service beneficiaries with AIS, 29.9% resided in the most vulnerable counties (SVI quartile 4), while 14.9% resided in counties with least social vulnerability (SVI quartile 1). AIS Medicare beneficiaries living in the most vulnerable counties had the highest proportions of adults aged 65 to 74 years, non-Hispanic Black or Hispanic, severe stroke at admission, a history of COVID-19, and more prevalent comorbidities. Compared to those living in least vulnerable counties, AIS Medicare beneficiaries living in most vulnerable counties had significantly higher all-cause mortality (adjusted HR: 1.11, 95% CI: 1.08-1.14). The pattern of association was largely consistent in subgroup analyses by age group, sex, and race and ethnicity.

**Conclusions:**

Higher social vulnerability levels were associated with increased all-cause mortality among AIS Medicare beneficiaries. To improve outcomes and address disparities, it may be important to focus efforts toward addressing social vulnerability.

There are longstanding inequities in stroke care and outcomes in the United States.^1^ During the COVID-19 pandemic, stroke mortality rates significantly increased, a reversal from decades of steady progress in the United States.^2^ While racial and ethnic disparities in stroke care and outcomes are well documented,^1^, ^2^, ^3^, ^4^ disparities in stroke mortality between Black and White adults widened during the COVID-19 pandemic, suggesting that the pandemic amplified existing inequities.^4^ The COVID-19 pandemic also highlighted the importance of community-level measures of social vulnerability in planning for emergent and nonemergent events. Studies suggested that communities with high levels of social vulnerability were associated with higher rates of SARS-CoV-2 (COVID-19) and mortality for several chronic conditions, including cardiovascular disease (CVD).^5^, ^6^, ^7^ To better understand the association between community-level measures of social determinants of health and stroke outcome during the COVID-19 pandemic, we linked the Centers for Disease Control and Prevention/Agency for Toxic Substances and Disease Registry’s Social Vulnerability Index (CDC/ATSDR SVI) to examine the distribution of risk factors across the SVI and explore the association between SVI and stroke mortality.

The CDC/ATSDR SVI was developed as a tool to identify communities that may be at high risk for natural disasters or disease outbreaks.^8^ Researchers extended the application of the SVI by examining its effects on CVD mortality^9^^,^^10^ and CVD-specific care outcomes,^11^^,^^12^ but its impact on stroke care outcomes remains understudied. Given that several studies have demonstrated the association between low socioeconomic status and worse stroke outcomes,^13^, ^14^, ^15^, ^16^ we hypothesize that all-cause mortality among those hospitalized for acute ischemic stroke (AIS) will be higher in adults living in the most socially vulnerable counties compared to the least vulnerable. This study aimed to examine the association between SVI quartile and all-cause mortality among Medicare fee-for-service (FFS) beneficiaries hospitalized with AIS between April 1, 2020, and December 31, 2021, with follow-up conducted until December 31, 2023.

## Materials and methods

We used Medicare inpatient monthly claims to identify Medicare FFS beneficiaries aged 65 years or older, hospitalized with incident AIS between April 1, 2020, and December 31, 2021 (COVID-19 pandemic period). AIS was defined as a hospital admission with a primary diagnosis of International Classification of Diseases-10th Revision-Clinical Modification (ICD-10-CM) code I63. If Medicare beneficiaries had multiple AIS hospitalizations during the study period, we selected the date of the first hospitalization. We excluded Medicare FFS beneficiaries with a pre-existing diagnosis of stroke (of any type, including transient ischemic attack) before the incident AIS hospitalizations, as per the Chronic Conditions Warehouse definition used by the Centers for Medicare and Medicaid Service (CMS).^17^ To identify cases of COVID-19, we accessed Medicare Part A (inpatient claims) and Part B (physician’s office claims) records, using ICD-10-CM code U07.1. We categorized AIS Medicare beneficiaries as having a history of COVID-19 if the first COVID-19 diagnosis date preceded the AIS admission date. For those with a history of COVID-19, hospitalization status was used to reflect the severity of the condition. We used National Institutes of Health Stroke Scale (NIHSS) scores (ICD-10-CM code: R29.7) to assess stroke severity.

The CDC/ATSDR SVI uses U.S. Census data to rank census tracts and counties on 16 social factors grouped into 4 themes: 1) socioeconomic status (below 150% poverty, unemployed, housing cost burden, no high school diploma, and no health insurance); 2) household characteristics (people aged ≥65 years, aged ≤17 years; civilians with a disability; single-parent households; and English language proficiency); 3) racial and ethnic minority status; and 4) housing type and transportation (multi-unit structures, mobile homes, crowding, no vehicle, and group quarters).^18^ The overall county-level SVI is a ranking percentile for each county that ranges from 0 (least vulnerable) to 1 (most vulnerable). We linked the county-level 2020 CDC/ATSDR SVI data to the Medicare data by county Federal Information Processing Standards code and classified SVI levels using county-level quartiles: quartile 1 (SVI: 0-0.25 [least vulnerable]), quartile 2 (SVI: 0.26-0.50), quartile 3 (SVI: 0.51-0.75), and quartile 4 (SVI: 0.76-1.0 [most vulnerable]). The final analytical study population had 176,123 Medicare FFS beneficiaries hospitalized with AIS.

Among Medicare beneficiaries with AIS, we calculated the median (IQR) and mean ± SEM of age and the percentage distribution of age group, sex, race and ethnicity, NIHSS score groups (0-9, 10-19, ≥20), history of COVID-19, percent of deaths during follow-up, and medical history of comorbidities at baseline, by SVI in quartiles. Comorbidities assessed included ischemic heart disease, hypertension, hypercholesterolemia, diabetes, atrial fibrillation, heart failure, chronic kidney disease, acute myocardial infarction, peripheral vascular disease, chronic obstructive pulmonary disease, and tobacco use. These comorbidities were based on the Chronic Conditions Warehouse definitions from CMS.^17^ Approximately 37% of Medicare beneficiaries with AIS had missing NIHSS scores, and we used multiple imputation to impute the missing values with 25 imputed datasets using PROC MI in SAS (SAS Institute).

We defined survival time as the number of months from the date of AIS hospitalization to the date of death or end of follow-up (December 31, 2023), whichever occurred first. To identify deaths that occurred in 2020 to 2021, we used the National Death Index data linked to Medicare data available through CMS. To identify deaths that occurred in 2022 to 2023, we used the Medicare Master Beneficiary Summary File, which contained the date of death based on the monthly enrollment status. We calculated all-cause mortality rates per 1,000 person-years overall and by age group, sex, and race and ethnicity groups. We performed Cox proportional hazards regression analyses to examine the association between SVI groups and all-cause mortality, adjusting for age group, sex, and race and ethnicity, and incorporated county-level cluster (Federal Information Processing Standards codes) as the random effect in the model. We conducted stratified Cox proportional hazards analysis by age group (66-74, 75-84, and ≥85 years), sex, and race and ethnicity (non-Hispanic White, non-Hispanic Black, Hispanic, and non-Hispanic other race). Additionally, the SVI was used as a continuous variable to examine its association with all-cause mortality after AIS hospitalizations. To explore this association, we incorporated a restricted cubic spline into our Cox proportional hazards models, using 3 knots at the 25th, 50th, and 75th percentiles of SVI.^19^ SAS, version 9.4 was used for the analyses, and a two-sided *P* value of <0.05 was considered statistically significant.

Since this study used deidentified Medicare claims data, it was considered not to be human subjects research and did not require review by an institutional review board. The Medicare beneficiaries’ data used in this study are not publicly available, and the authors cannot share the data because of the Data Use Agreement with CMS. However, Medicare data are available for purchase from CMS following a data use request.

## Results

There were 176,123 Medicare FFS beneficiaries hospitalized with the first (or incident) AIS as the primary diagnosis between April 1, 2020, and December 31, 2021. Among these, 14.9% resided in counties categorized as least vulnerable (SVI quartile 1) and 29.9% resided in counties categorized as most vulnerable (SVI quartile 4), while 26.0% and 29.2% resided in counties with SVI quartiles of 2 and 3, respectively. The median age at AIS was 78.3 years; 45.1% were men; 82.1% were non-Hispanic White; and 6.3% had a history of COVID-19 before AIS (Table 1).

**Table 1:** Demographic and Clinical Information by SVI Quartiles

	Overall or Statistics (N = 176,123)	SVI^a^				
		Quartile 1 (Least Vulnerable) (n = 26,240)	Quartile 2 (n = 45,816)	Quartile 3 (n = 51,448)	Quartile 4 (Most Vulnerable) (n = 52,619)	*P* Value^b^
Age at AIS						
Median	78.3	78.6	78.6	78.2	77.8	
IQR	72.0-85.7	72.3-85.7	72.4-85.5	72.0-85.2	71.6-84.8	
Mean ± SEM	79.0 ± 0.02	79.3 ± 0.05	79.3 ± 0.04	79.0 ± 0.04	78.6 ± 0.04	<0.0001
Age in groups						
65-74	54,483 (30.9)	7,707 (29.4)	13,444 (29.3)	16,009 (31.1)	17,323 (32.9)	
75-84	68,453 (38.9)	10,238 (39.0)	18,027 (39.3)	19,983 (38.8)	20,205 (38.4)	
≥85	53,187 (30.2)	8,295 (31.6)	14,345 (31.3)	15,456 (30.0)	15,091 (28.7)	<0.0001
Male	79,450 (45.1)	12,073 (46.0)	20,512 (44.8)	23,260 (45.2)	23,605 (44.9)	0.007
Race and ethnicity						
Non-Hispanic White	144,528 (82.1)	24,495 (93.3)	40,478 (88.3)	43,187 (83.9)	36,368 (69.1)	
Non-Hispanic Black	16,353 (9.3)	860 (3.3)	2,517 (5.5)	4,210 (8.2)	8,766 (16.7)	
Hispanic	8,226 (4.7)	358 (1.4)	1,115 (2.4)	1,894 (3.7)	4,859 (9.2)	
Non-Hispanic other	7,016 (4.0)	527 (2.0)	1,706 (3.7)	2,157 (4.2)	2,626 (5.0)	<0.0001
NIHSS scores in group						
0-9	135,336 (76.8)	20,582 (78.4)	35,522 (77.5)	39,604 (77.0)	39,628 (75.3)	
10-19	25,860 (14.7)	3,592 (13.7)	6,568 (14.3)	7,495 (14.6)	8,205 (15.6)	
≥20	14,927 (8.5)	2,066 (7.9)	3,726 (8.1)	4,349 (8.5)	4,786 (9.1)	<0.0001
COVID-19 status						
History of hospitalized COVID-19	5,230 (3.0)	687 (2.6)	1,275 (2.8)	1,524 (3.0)	1,744 (3.3)	
History of nonhospitalized COVID-19	5,894 (3.3)	789 (3.0)	1,368 (3.0)	1,699 (3.3)	2,038 (3.9)	
No COVID-19	164,999 (93.7)	24,764 (94.4)	43,173 (94.2)	48,225 (93.7)	48,837 (92.8)	<0.0001
Death as of follow-up on December 31, 2023	74,371 (42.2)	10,837 (41.3)	18,988 (41.4)	21,592 (42.0)	22,954 (43.6)	<0.0001
Comorbidities						
Ischemic heart disease	85,831 (48.7)	12,175 (46.4)	22,062 (48.2)	25,025 (48.6)	26,569 (50.5)	<0.0001
Hypertension	143,606 (81.5)	20,924 (79.7)	37,263 (81.3)	42,082 (81.8)	43,337 (82.4)	<0.0001
Hypercholesterolemia	134,553 (76.4)	19,924 (75.9)	35,336 (77.1)	39,354 (76.5)	39,939 (75.9)	<0.0001
Diabetes	72,998 (41.4)	9,778 (37.3)	18,312 (40.0)	20,804 (40.4)	24,104 (45.8)	<0.0001
Atrial fibrillation	34,091 (19.4)	5,188 (19.8)	9,118 (19.9)	10,011 (19.5)	9774 (18.6)	<0.0001
Heart failure	49,827 (28.3)	6,769 (25.8)	12,495 (27.3)	14,177 (27.6)	16,386 (31.1)	<0.0001
Chronic kidney disease	73,091 (41.5)	10,312 (39.3)	18,590 (40.6)	21,264 (41.3)	22,925 (43.6)	<0.0001
Acute myocardial infarction	11,093 (6.3)	1,740 (6.6)	2,920 (6.4)	3,244 (6.3)	3,189 (6.1)	<0.0001
Peripheral vascular disease	28,375 (16.1)	4,109 (15.7)	7,143 (15.6)	8,116 (15.8)	9,007 (17.1)	0.02
COPD	45,539 (25.9)	6,241 (23.8)	11,491 (25.1)	13,635 (26.5)	14,172 (26.9)	<0.0001
Tobacco use	15,559 (8.8)	2,132 (8.1)	3,834 (8.4)	4,751 (9.2)	4,842 (9.2)	<0.0001

Values are n (%) unless otherwise indicated.

AIS = acute ischemic stroke; COPD = chronic obstructive pulmonary disease; CDC/ATSDR = Centers for Disease Control and Prevention/Agency for Toxic Substances and Disease Registry; IQR = interquartile range; NIHSS = National Institutes of Health Stroke Scale; SVI = Social Vulnerability Index.

a SVI quartiles were based on county-level 2020 CDC/ATSDR SVI data.

b The differences between continuous variables across SVI levels were examined using the Kruskal-Wallis test, and the differences between categorical variables across SVI levels were examined using chi-square test.

Significant differences in demographic and clinical features were observed by SVI quartiles. There was a significant decrease in median age at AIS, a decrease in the proportion of male, and an increase in the proportion of non-Hispanic Black or Hispanic, from SVI quartile 1 to quartile 4 (Table 1). Compared to Medicare beneficiaries living in least vulnerable counties, those living in most vulnerable counties were younger (median age 77.8 vs 78.6 years); more likely to be non-Hispanic Black (16.7% vs 3.3%) or Hispanic (9.2% vs 1.4%); presented with more severe stroke at admission (9.1% NIHSS score ≥20 vs 7.9%); had a higher prevalence of COVID-19 history (7.2% vs 5.6%); and had a higher prevalence of most comorbidities. The proportion of deaths (as of December 31, 2023) was higher in those living in the most vulnerable counties (43.6%) compared to the least vulnerable (41.3%) (Table 1).

The all-cause mortality rates per 1,000 person-year increased from SVI quartile 1 at 207 (95% CI: 203-211), to 227 (95% CI: 224-230) in SVI quartile 4 (Table 2). Analyses stratified by age group showed the all-cause mortality rates increased with age, and analysis stratified by sex showed higher rates among females compared to males. After adjusting for age, sex, and race and ethnicity, Medicare beneficiaries living in the most vulnerable counties (SVI quartile 4) were more likely to die than those living in the least vulnerable counties (SVI quartile 1) (adjusted hazard ratio (HR): 1.11; 95% CI: 1.08-1.14) (Table 2). Among Medicare beneficiaries hospitalized with AIS, the patterns of risk of all-cause mortality across SVI quartiles were generally consistent across age groups, sex, and race and ethnicity.

**Table 2:** Adjusted Proportional HR (95% CI) for All-Cause Mortality

	SVI^a^			
	Quartile 1 (Least Vulnerable)	Quartile 2	Quartile 3	Quartile 4 (Most Vulnerable)
Overall				
Number of deaths (person-years)	10,837 (52,328)	18,988 (908,389)	21,592 (101,003)	22,954 (101,220)
Death rate per 1,000 person-years	207 (203-211)	209 (206-212)	214 (211-217)	227 (224-230)
Adjusted HR (95% CI)^b^	Reference	1.01 (0.99-1.04)	1.04 (1.02-1.07)	1.11 (1.08-1.14)
Subgroup analyses				
Age 65-74 y				
Number of deaths (person-years)	2,115 (17,384)	3,908 (29,773)	4,856 (35,008)	5,644 (37,084)
Death rate per 1,000 person-years	122 (117-127)	131 (127-135)	139 (135-143)	152 (148-156)
Adjusted HR (95% CI)^b^	Reference	1.07 (1.02-1.13)	1.12 (1.07-1.18)	1.22 (1.16-1.28)
Age 75-84 y				
Number of deaths (person-years)	3,720 (21,570)	6,511 (38,036)	7,363 (41,617)	7,954 (41,082)
Death rate per 1,000 person-years	172 (167-178)	171 (167-175)	177 (173-181)	194 (189-198)
Adjusted HR (95% CI)^b^	Reference	0.99 (0.95-1.04)	1.02 (0.98-1.06)	1.11 (1.06-1.15)
Age >85 y				
Number of deaths (person-years)	5,002 (13,374)	8,659 (23,030)	9,373 (24,378)	9,356 (23,054)
Death rate per 1,000 person-years	374 (364-385)	372 (364-380)	384 (377-392)	406 (398-414)
Adjusted HR (95% CI)^b^	Reference	0.99 (0.96-1.03)	1.02 (0.98-1.06)	1.06 (1.02-1.10)
Male				
Number of deaths (person-years)	4,705 (24,745)	8,072 (41,828)	9,345 (46,762)	9,945 (46,362)
Death rate per 1,000 person-years	190 (185-196)	193 (189-197)	200 (196-204)	215 (210-219)
Adjusted HR (95% CI)^b^	Reference	1.01 (0.97-1.05)	1.05 (1.01-1.09)	1.14 (1.09-1.18)
Female				
Number of deaths (person-years)	6,132 (27,583)	10,916 (49,011)	12,247 (54,241)	13,009 (54,859)
Death rate per 1,000 person-years	222 (217-228)	223 (219-227)	226 (222-230)	237 (233-241)
Adjusted HRs^b^	Reference	1.01 (0.98-1.04)	1.03 (1.00-1.06)	1.09 (1.05-1.12)
Non-Hispanic White				
Number of deaths (person-years)	10,147 (48,759)	16,924 (79,957)	18,208 (84,628)	15,854 (69,923)
Death rate per 1,000 person-years	208 (204-212)	212 (208-215)	215 (212-218)	227 (223-230)
Adjusted HR (95% CI)^b^	Reference	1.02 (0.99-1.04)	1.04 (1.02-1.07)	1.10 (1.07-1.13)
Non-Hispanic Black				
Number of deaths (person-years)	352 (1,740)	1,007 (5,061)	1,768 (8,308)	3,925 (16,839)
Death rate per 1,000 person-years	202 (182-225)	199 (187-212)	213 (203-223)	233 (226-241)
Adjusted HR (95% CI)^b^	Reference	0.99 (0.88-1.12)	1.04 (0.93-1.17)	1.15 (1.03-1.28)
Hispanic				
Number of deaths (person-years)	145 (735)	403 (2,336)	724 (3,833)	2,056 (9,463)
Death rate per 1,000 person-years	197 (168-232)	173 (156-190)	189 (176-203)	217 (208-227)
Adjusted HR (95% CI)^b^	Reference	0.88 (0.73-1.07)	0.94 (0.79-1.13)	1.07 (0.90-1.27)
Non-Hispanic Other				
Number of deaths (person-years)	193 (1,094)	654 (3,486)	892 (4,234)	1,119 (4,995)
Death rate per 1,000 person-years	176 (153-203)	188 (174-203)	211 (197-225)	224 (211-238)
Adjusted HR (95% CI)^b^	Reference	0.98 (0.84-1.16)	1.11 (0.94-1.29)	1.19 (1.02-1.38)

CDC/ATSDR = Centers for Disease Control and Prevention/Agency for Toxic Substances and Disease Registry; FIPS = Federal Information Processing Standards; SVI = Social Vulnerability Index.

a SVI quartiles were based on county-level 2020 CDC/ATSDR SVI data.

b HRs were estimated using Cox proportional hazards models that incorporated county FIPS codes as the random factor and adjusted for age group, sex, and race and ethnicity.

**Figure 1:**
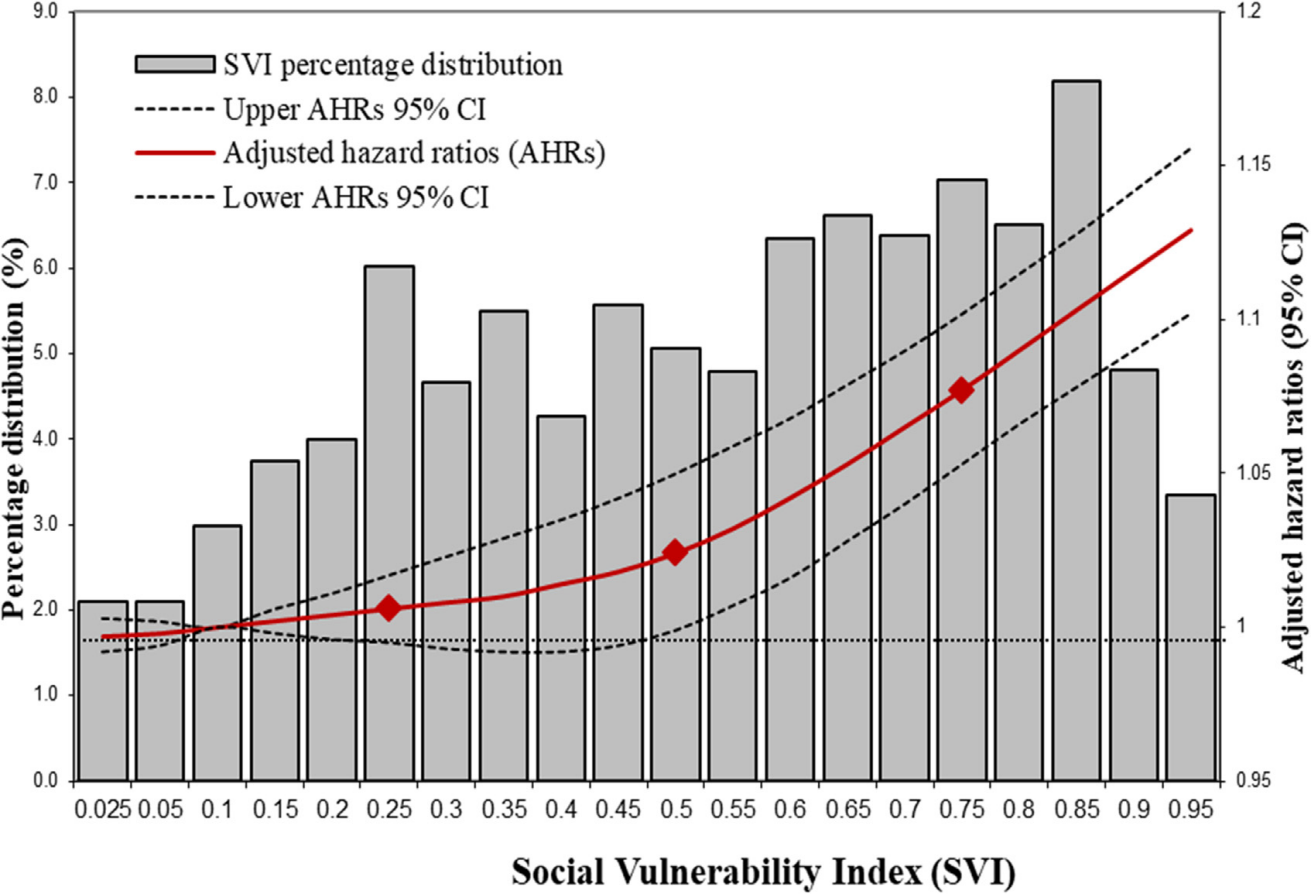
SVI Distribution and Adjusted HRs for All-Cause Mortality SVI = Social Vulnerability Index.

As a continuous measure, the SVI showed a nonlinear relationship with all-cause mortality among Medicare beneficiaries hospitalized with AIS, indicating stronger association with all-cause mortality above the 50th percentile of SVI (Figure 1).

**Central Illustration:**
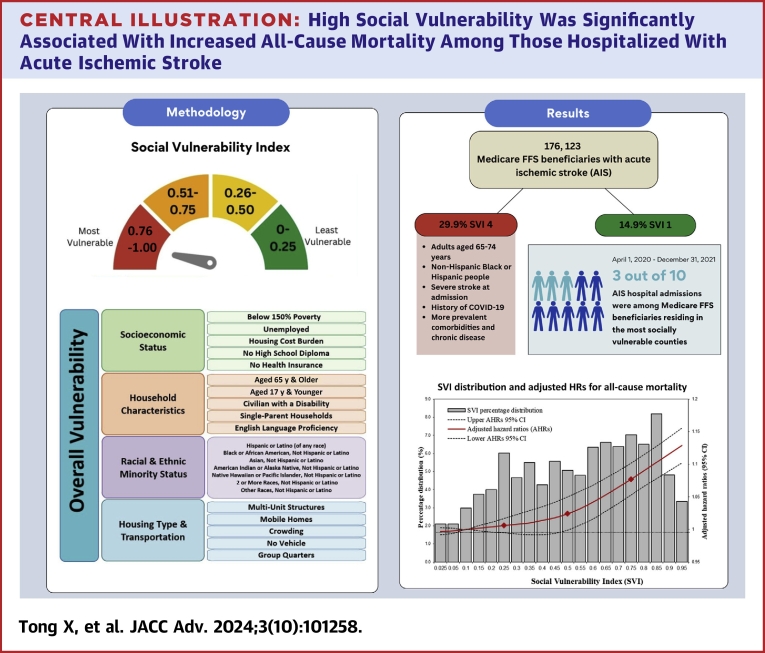
High Social Vulnerability Was Significantly Associated With Increased All-Cause Mortality Among Those Hospitalized With Acute Ischemic Stroke SVI = Social Vulnerability Index.

## Discussion

About 3 out of 10 AIS admissions were among Medicare beneficiaries residing in most socially vulnerable counties, with disparities across SVI level by race and ethnicity, stroke severity, and presence of comorbidities. High social vulnerability was significantly associated with increased all-cause mortality among those hospitalized with AIS, independent of age, sex, and race and ethnicity.

Our study showed that Medicare FFS beneficiaries hospitalized with AIS residing in the most vulnerable counties (SVI quartile 4) had the highest proportions of adults aged 65 to 74 years and non-Hispanic Black or Hispanic adults. These beneficiaries also had more severe NIHSS scores and the highest prevalence of many comorbidities, including ischemic heart disease, hypertension, diabetes, heart failure, chronic kidney disease, chronic obstructive pulmonary disease, and tobacco use. Some of these findings may be due to overlap of the SVI’s community-level components with individual-level factors. The specific inclusion of a racial and ethnic minority status theme within the CDC/ATSDR SVI also likely contributes to the disparities by race and ethnicity that we observed. Racial and ethnic disparities that we identified in Medicare beneficiaries hospitalized with AIS residing in the most vulnerable counties are consistent with other studies using data at different geographic levels. Studies examining state-level data have similarly shown that people living in states characterized by the highest SVI scores are more likely to be Black or Hispanic and have a higher burden of comorbidities.^20^ Prior research using neighborhood-level data also demonstrated a higher prevalence of cardiovascular risk factors, including hypertension, diabetes, and smoking, in areas characterized by higher SVI.^21^

Our study showed that AIS Medicare beneficiaries residing in the most vulnerable counties faced an increased all-cause mortality risk of about 11% in comparison to those living in least vulnerable counties, and this association was consistent across age groups, sex, and race and ethnicity. This association between higher social vulnerability and higher all-cause mortality risk among those hospitalized with AIS was consistent with prior studies. Studies have indicated that living in neighborhoods and counties characterized as low socioeconomic status and more social vulnerabilities is independently associated with increased risk of CVD-related death^10^ and higher premature CVD mortality, including stroke mortality.^9^ However, contrary to our study findings, some studies did identify differences by demographic characteristics in related associations.^9^^,^^22^ One study showed the association between SVI quartiles and stroke mortality was significant only among non-Hispanic Black adults,^9^ and another study showed the association between individual and community-level social determinants of health and stroke incidence varied by age group.^22^ Reasons for differences in findings may be due to the study population among people aged 18 to 64 years^9^ or aged 45 years and older,^22^ as compared to Medicare beneficiaries in our study. Future studies may wish to further examine the independent and combined effects of demographic characteristics and community-level social vulnerability measures on health risk.

The COVID-19 pandemic disproportionately affected socioeconomically disadvantaged population in the United States, with higher COVID-19 incidence associated with higher SVI.^23^ Our study of Medicare beneficiaries found the highest prevalence of a history of COVID-19 among patients with the highest social vulnerability, which aligned with previous findings that the counties with the highest incidence of COVID-19 cases and worse outcomes were those facing heightened social vulnerability.^23^, ^24^, ^25^

There are several possible factors that may contribute to higher mortality for Medicare beneficiaries in high SVI counties, and these may be contributory factors to pervasive social inequities.^26^ First, the duration between the onset of a stroke and hospital arrival is a crucial factor for prompt acute stroke care and significantly influencing stroke outcomes.^27^ Several studies found social inequities in prehospital stroke care, revealing patients from a lower socioeconomic neighborhood had significant delays from the emergency call to the hospital arrival compared with patients from a higher socioeconomic neighborhood.^27^ Furthermore, counties categorized as the most vulnerable (quartile 4) had significantly longer travel time to access advanced stroke care centers compared to their less vulnerable counterparts (quartile 1), based on the Minority Status and Language component of SVI.^28^ Second, the lack of access to health care for managing CVD risk factors and comorbidities might partly explain the higher mortality observed in the most vulnerable SVI counties. A cross-sectional study using data from the Behavioral Risk Factor Surveillance System demonstrated the association between SVI and health care access, reporting a higher prevalence of difficulty accessing health care services among U.S. states with greater social vulnerability.^20^ From the perspective of primary prevention of CVD, areas with high levels of social vulnerability may lack resources for heart-healthy environments, such as places to buy healthy food and exercise in a safe environment.^29^^,^^30^

Understanding the role of county-level social vulnerability, which ranks counties on several social determinants of health factors, may help to explain and address disparities in stroke. The American Heart Association released a scientific statement documenting the substantial roles of social determinants of health on the incidence, treatment, and outcomes of CVD.^31^ The American Stroke Association and National Institute of Neurological Disorders and Stroke established the Health Equity and Actionable Disparities in Stroke symposium in 2020 focusing on research in identifying inequities in cerebrovascular disease and calling for community-engaged strategies to reduce inequities in stroke.^1^ The pandemic brought to light the large role played by social determinants of health in the nation’s lives and health.^32^ It is important to address these factors with coordinated and multisectoral strategies to achieve health equity and improve health outcomes across communities.^32^ Finally, leveraging insights from measures like the CDC/ATSDR SVI may help future research identify the vulnerable populations and implement targeted interventions to ultimately reduce health disparities related to CVD.^33^^,^^34^

Our study has several limitations. First, AIS hospitalizations, COVID-19 diagnoses, and all-cause mortality were based on administrative records and limited to Medicare FFS beneficiaries aged ≥65 years. We may have omitted some beneficiaries with diagnosed COVID-19, diagnosed AIS, or incorrect diagnosis dates of COVID-19 due to our use of Medicare administrative data. Second, our study included the early phase COVID-19 patients (April 1, 2020 to December 31, 2021). There is no detailed information of COVID-19 variants in Medicare data for the early phase of COVID-19, and we were unable to stratify the analysis by strain of COVID-19. Third, NIHSS scores were based on ICD-10 codes, which may be inaccurate. In addition, 37% of Medicare beneficiaries hospitalized with AIS had NIHSS scores missing and had to be imputed. Fourth, it is possible that some of the deaths may not have been recorded in Medicare beneficiary enrollment files by December 31, 2023, and we might underestimate all-cause mortality. Fifth, we linked county level SVI data, representing large geographic areas with possible substantial heterogeneity in community composition, which could contribute to limited generalizability to subcounty vulnerability estimates. Lastly, the findings based on FFS beneficiaries may not be generalizable to Medicare patients covered under health maintenance organization plans due to possible differences in beneficiary characteristics with the 2 types of coverage.

## Conclusions

Medicare beneficiaries with AIS living in the most vulnerable counties had a higher mortality, regardless of age, sex, and race and ethnicity. Beneficiaries residing in the most socially vulnerable counties were more likely to be non-Hispanic Black or Hispanic adults and have more severe strokes and more prevalent comorbidities. To improve AIS outcomes and address disparities, it may be important to focus efforts on the most vulnerable counties and ensure that preventive strategies are tailored to meet the needs of those communities.

## Funding support and author disclosures

The authors have reported that they have no relationships relevant to the contents of this paper to disclose.
